# Phenotypic Robustness and the Assortativity Signature of Human Transcription Factor Networks

**DOI:** 10.1371/journal.pcbi.1003780

**Published:** 2014-08-14

**Authors:** Dov A. Pechenick, Joshua L. Payne, Jason H. Moore

**Affiliations:** 1 Computational Genetics Laboratory, Dartmouth College, Hanover, New Hampshire, United States of America; 2 Institute of Evolutionary Biology and Environmental Studies, University of Zurich, Zurich, Switzerland; Ohio University, United States of America

## Abstract

Many developmental, physiological, and behavioral processes depend on the precise expression of genes in space and time. Such spatiotemporal gene expression phenotypes arise from the binding of sequence-specific transcription factors (TFs) to DNA, and from the regulation of nearby genes that such binding causes. These nearby genes may themselves encode TFs, giving rise to a transcription factor network (TFN), wherein nodes represent TFs and directed edges denote regulatory interactions between TFs. Computational studies have linked several topological properties of TFNs — such as their degree distribution — with the robustness of a TFN's gene expression phenotype to genetic and environmental perturbation. Another important topological property is assortativity, which measures the tendency of nodes with similar numbers of edges to connect. In directed networks, assortativity comprises four distinct components that collectively form an assortativity signature. We know very little about how a TFN's assortativity signature affects the robustness of its gene expression phenotype to perturbation. While recent theoretical results suggest that increasing one specific component of a TFN's assortativity signature leads to increased phenotypic robustness, the biological context of this finding is currently limited because the assortativity signatures of real-world TFNs have not been characterized. It is therefore unclear whether these earlier theoretical findings are biologically relevant. Moreover, it is not known how the other three components of the assortativity signature contribute to the phenotypic robustness of TFNs. Here, we use publicly available DNaseI-seq data to measure the assortativity signatures of genome-wide TFNs in 41 distinct human cell and tissue types. We find that all TFNs share a common assortativity signature and that this signature confers phenotypic robustness to model TFNs. Lastly, we determine the extent to which each of the four components of the assortativity signature contributes to this robustness.

## Author Summary

The cells of living organisms do not concurrently express their entire complement of genes. Instead, they regulate their gene expression, and one consequence of this is the potential for different cells to adopt different stable gene expression patterns. For example, the development of an embryo necessitates that cells alter their gene expression patterns in order to differentiate. These gene expression phenotypes are largely robust to genetic mutation, and one source of this robustness may reside in the network structure of interacting molecules that underlie genetic regulation. Theoretical studies of regulatory networks have linked network structure to robustness; however, it is also necessary to more extensively characterize real-world regulatory networks in order to understand which structural properties may be biologically meaningful. We recently used theoretical models to show that a particular structural property, degree assortativity, is linked to robustness. Here, we measure the assortativity of human regulatory networks in 41 distinct cell and tissue types. We then develop a theoretical framework to explore how this structural property affects robustness, and we find that the gene expression phenotypes of human regulatory networks are more robust than expected by chance alone.

## Introduction

Cells are capable of expressing specific subsets of their gene complement in a coordinated fashion, leading to stable gene expression phenotypes. Such gene expression phenotypes may, for example, characterize the differentiation stage of a cell [1] or a cell's ability to thrive under specific environmental conditions [2]. The spatiotemporal regulation of gene expression is thus an important means by which cells cope with their surroundings, and is also instrumental in the processes driving organismal development [3].

Transcription factors (TFs) constitute one means by which this regulation is carried out. TFs are proteins that bind DNA to regulate the expression of their target genes. Since some of the targets are themselves TFs, the resulting cross-regulation forms a transcription factor network (TFN). In a TFN, an edge 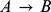 exists if the protein product of TF-*A* regulates the expression of the gene that encodes TF-*B* [4]. TFNs are responsible for metazoan developmental programs, such as the development of skeletal muscle [5] and the formation of the retina [6]. They are also involved in generating oscillatory gene expression patterns, such as those that drive the cell cycle [7] and the mammalian circadian clock [8]. TFNs have been studied across a range of organisms, including the bacterium *Escherichia coli* [9], the yeast *Saccharomyces cerevisiae* [10], the sea urchin *Strongylocentrotus purpuratus* [11], and human [12], [13]. The characterization of transcriptional regulation as TFNs has enabled researchers to implement a host of analytical tools from network science. In particular, the topology of TFNs has been the subject of work seeking a greater understanding of how the structure of a TFN affects its function [14], and likewise how evolution may [15] or may not [16] mold its structural properties.

In conjunction with such analyses, there have been a number of theoretical studies linking the topology of TFN models with the robustness of their gene expression patterns (phenotypes). For example, both increased modularity [17] and a heavy-tailed degree distribution [18] have been shown to confer robustness to genetic mutation and environmental noise. Furthermore, evolutionary processes can alter the robustness of a TFN model through incremental changes in topology [19].

Another topological property that has been linked to the robustness of TFN models is degree assortativity. This is a measure of the tendency for nodes with similar numbers of connections to themselves be connected, where a strong tendency approaches the value of 1 and the opposite tendency approaches the value -1 [20]. Theoretical work has shown that TFN models with increased assortativity exhibit increased robustness to both mutation in *cis*-regulatory sites [21] and to gene duplication [22]. This occurs because increased assortativity may either shrink the average size of nested subgraphs within the network (in-components) [21] or increase the average number of regulatory links that separate TFs (characteristic path length) [23], both of which tend to dampen the phenotypic effects of mutations. This earlier work focused exclusively on the assortativity of outgoing connections, referred to as out-out assortativity, and thus the findings suggest that TFNs are more robust when for some edge 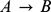 it is frequently the case that TFs *A* and *B* regulate a similar number of targets. However, because TFNs are directed networks where each TF may have both incoming and outgoing connections, there are a total of four types of degree assortativity that may be measured. The other three types are referred to as out-in, in-out, and in-in assortativity. Along with out-out assortativity, they convey topological information about which TFs regulate which other TFs, and it is an open question as to whether these types of assortativity influence the robustness of TFNs to genetic perturbation.

These four types of assortativity have been measured for a number of real-world directed networks, including online and social networks, food webs, and linguistic networks [24], revealing two striking trends. First, assortativity was found to deviate from the null expectation in a manner specific to the type of networked system being considered. Second, discipline-specific methods for the modeling of these real-world networks did not always recapitulate the observed assortativity, implying a gap in the understanding of why certain networks are structured the way they are. It is therefore possible that the four types of assortativity may affect the dynamical properties of networked systems, such as TFNs. However, little is currently known about the assortativity of real-world TFNs.

In this study, we calculate the assortativity of 41 recently elaborated human cell-specific TFNs [13]. We assess to what extent the four assortativity values differ from those expected at random, resulting in an assortativity signature for each TFN. We then investigate the effects of common elements of these signatures on the phenotypic robustness of TFN models to genetic perturbation. Finally, we create a suite of artificial signatures to further explore how the four different components of assortativity contribute to phenotypic robustness.

## Results

### The Data

**Figure 1:**
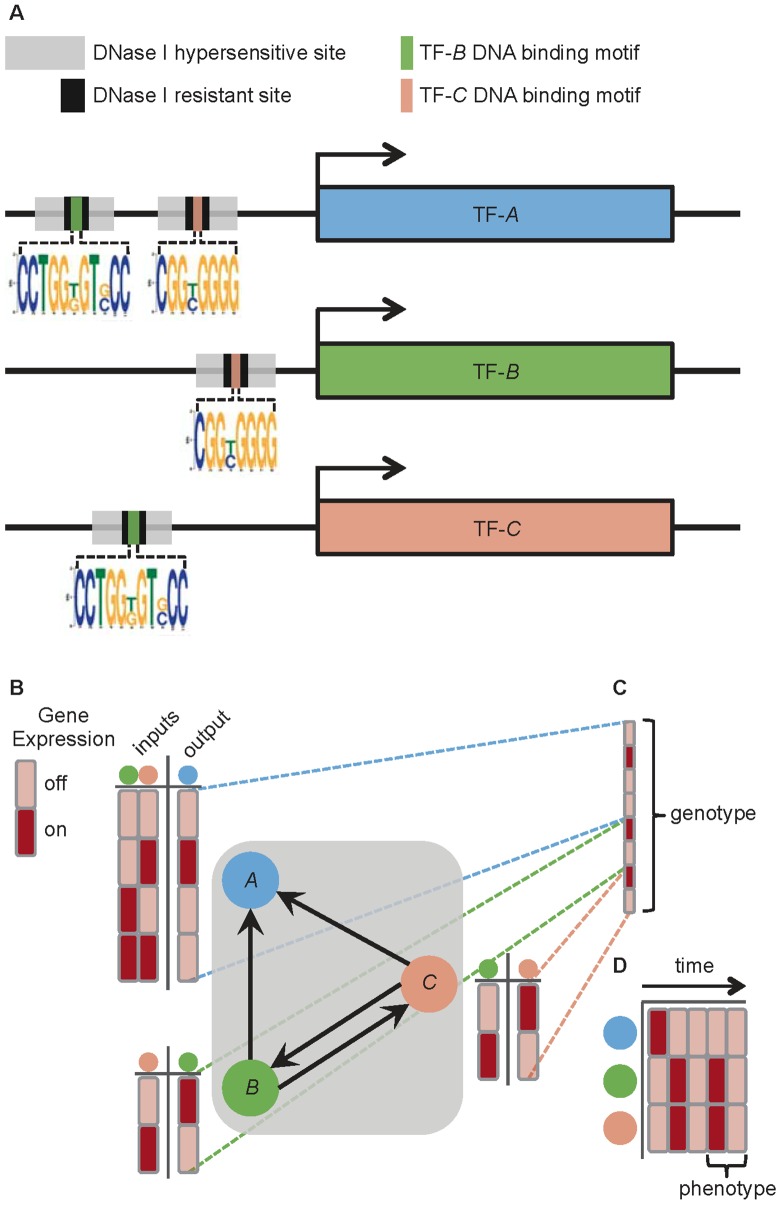
Constructing human transcription factor networks (TFNs) from genome-wide DNase I hypersensitivity profiles and motif analysis. (A) The *cis*-regulatory regions of DNA directly upstream of the genes encoding hypothetical TFs (TF-*A*, TF-*B*, and TF-*C*) contain DNase I hypersensitive sites that are accessible to protein binding. The evidence for binding events are the DNase I resistant footprints within the hypersensitive sites. Although the identity of the protein that leaves a footprint is not directly observed, the recognition of a TF-specific DNA binding motif enables the inference of which TF is bound at that footprint. In this hypothetical example, binding sites for both TF-*B* and TF-*C* are found within footprints in DNase I hypersensitive sites upstream of the gene encoding TF-*A*. Therefore, TF-*B* and TF-*C* are inferred to be bound upstream of the gene for TF-*A*. Likewise, TF-*B* and TF-*C* are bound upstream of each other's genes. (B) These inferred binding events are represented as directed edges in the TFN, *i.e.*, 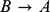, 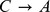, 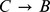, and 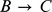. The dynamics of this TFN can be modeled using a Boolean framework, as follows. The state of each TF is considered either off or on at any given time, and regulatory rules (shown here as truth tables) dictate the future states of TFs based on their current states. (C) The regulatory rules for the entire TFN model is its genotype. (D) The states of all the TFs in the TFN model at a particular time is referred to as its configuration at that time. Given an initial configuration, the configuration at each subsequent time point is updated according to the genotype. The TFN model has a finite number of possible configurations, and the genotype synchronously and deterministically updates one to the next. Therefore, the TFN model inevitably encounters an indefinitely repeating cycle of configurations, which represents the model's phenotype.

In order to address the question of whether human regulatory networks have nonrandom assortativity, we chose to examine the topology of 41 human cell-specific transcription factor networks (TFNs) [13]. These TFNs were generated through genomic footprinting [25]. This approach combines DNase I sensitivity analysis with known TF-specific DNA binding motifs, and thus enables the inference of a large number of specific TF-DNA binding events. The 41 TFNs contain between 485 and 526 TFs and between 8,821 and 18,348 directed edges (Table S1), where an edge is defined as the inferred binding of a specific TF within the *cis*-regulatory region of a gene encoding another TF. Inferring the identity of the bound TF is made possible through the recognition of known TF binding motifs. As an example, if there were evidence that the *cis*-regulatory region of the gene encoding TF-*A* is bound by the protein TF-*B*, then a directed edge from TF-*B* to TF-*A* would be included in the TFN (Fig. 1).

### Human Transcription Factor Networks Possess a Distinct Assortativity Signature

**Figure 2:**
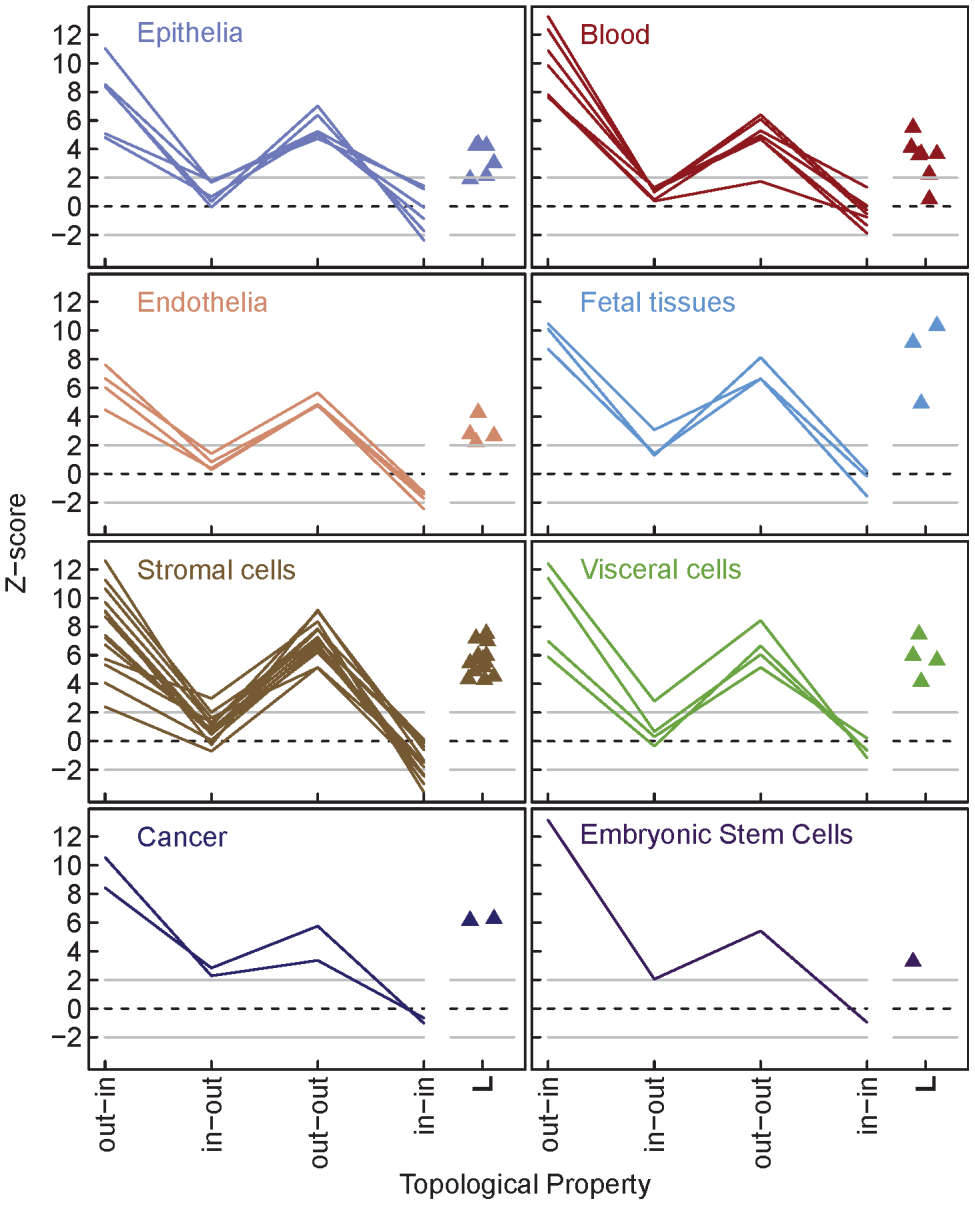
Human transcription factor networks (TFNs) share a common assortativity signature. Z-scores for all four types of degree-assortativity (out-in, in-out, out-out, and in-in) are plotted for each of the 41 human TFNs, grouped in panels by cell type [13]. The colored lines connecting the four scores are provided as a visual representation of the assortativity signature of each TFN. Z-scores for characteristic path length (*L*) are plotted separately from the assortativity signature as triangles. Z-scores for each TFN were generated by comparing the observed TFN to a null distribution of 1000 randomly rewired TFNs (see Methods). A Z-score greater than 2 or less than -2 is considered significant.

We first computed each of the four assortativity values for all 41 human TFNs, and converted these values into their corresponding Z-scores [24]. Each Z-score is defined as the difference between the observed assortativity value for the TFN and the mean of its null distribution, scaled by the standard deviation of its null distribution (see Methods). The advantage of using Z-scores instead of raw assortativity values is that they are directly comparable across different TFNs, and convey the extent to which assortativity deviates from the null expectation. The assortativity Z-scores of the 41 human TFNs revealed a distinct signature (Fig. 2). There are two notable features of this signature, which we will refer to as the *human signature*. First, few of the TFNs appear nonrandom with respect to in-out (7 of 41 TFNs) or in-in (8 of 41 TFNs) assortativity. In contrast, nearly all the TFNs display greater-than-expected out-in (40 of 41 TFNs) and out-out assortativity (40 of 41 TFNs).

We then investigated whether the increased out-out assortativity of human TFNs was associated with other nonrandom topological properties, in particular average in-component (IC) size and characteristic path length (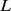). The IC of a TF 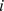 is the set of TFs that directly or indirectly regulate 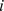, and average IC size has been shown to be negatively correlated with out-out assortativity in TFN models [21]. However, here we rarely observed smaller-than-expected average IC size in the human TFNs (5 of 41 TFNs, 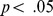 computed from the null distributions; see Methods) despite their increased out-out assortativity. In contrast, 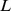 (*i.e.*, the average length of the shortest directed paths between all pairs of TFs) is positively correlated with out-out assortativity in TFN models, specifically when average IC size is not smaller-than-expected [23]. In line with this finding, we observed greater-than-expected 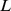 in almost all of the human TFNs (39 of 41 TFNs, Fig. 2).

### The Human Assortativity Signature Confers Robustness to Dense Transcription Factor Network Models

Since the human TFNs possess topological properties that include increased out-out assortativity and increased 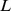, we asked whether they also display increased phenotypic robustness in response to mutation. To address this, we created random Boolean networks [26] as TFN models to approximate the human TFNs (Fig. 1B; see Methods). Due to the computational burden of simulating individual phenotypes for TFN models as large as the human TFNs (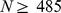), and the infeasibility of estimating robustness for multiple phenotypes over thousands of large model networks, we constructed more manageable TFN models with 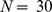. TFN models of this size are: 1) small enough to provide computational tractability, 2) large enough to uncover trends between assortativity and model dynamics [21], and 3) recapitulate the same trends seen in models with hundreds of nodes [22]. Although much smaller than the human TFNs, the models were constructed with two important characteristics of the human TFNs in mind. First, these TFN models incorporated the human assortativity signature, taken as the average of all 41 signatures observed for the human TFNs (Fig. 2). Second, their average IC sizes were constrained to what would be expected by random chance, since very few (5 of 41) human TFNs deviated from the null expectation (Fig. 2). These two requirements produced TFN models with above average 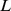, as expected theoretically [23] and observed in the human TFNs (Fig. 2).

We then estimated the phenotypic robustness, here referred to simply as robustness, of the TFN models according to Pechenick et al. [21] (see Methods). In brief, a random walk was conducted in the space of possible genotypes for each TFN model, where the genotype is the set of regulatory rules that governs the timing of TF expression in the model (Fig. 1C). This timing results in a stable pattern of gene expression, which is regarded as the phenotype of the TFN model (Fig. 1D). A single point mutation within the genotype serves as a step in the random walk, and corresponds to a perturbation in a gene's *cis*-regulatory region, such as a single nucleotide change that alters the affinity of the TF that binds that region [27]. In our model, this translates to changing a single, randomly chosen element of the genotype (from 0 to 1, or vice versa) without modifying network topology. Such a change to a network's regulatory logic may or may not affect the gene expression phenotype. If the mutated genotype does not alter the phenotype, then the step is considered successful and the walk proceeds from the new genotype. Not all steps are successful (*i.e.*, some steps perturb the phenotype), and the proportion of successful steps serves as a measure of phenotypic robustness.

**Figure 3:**
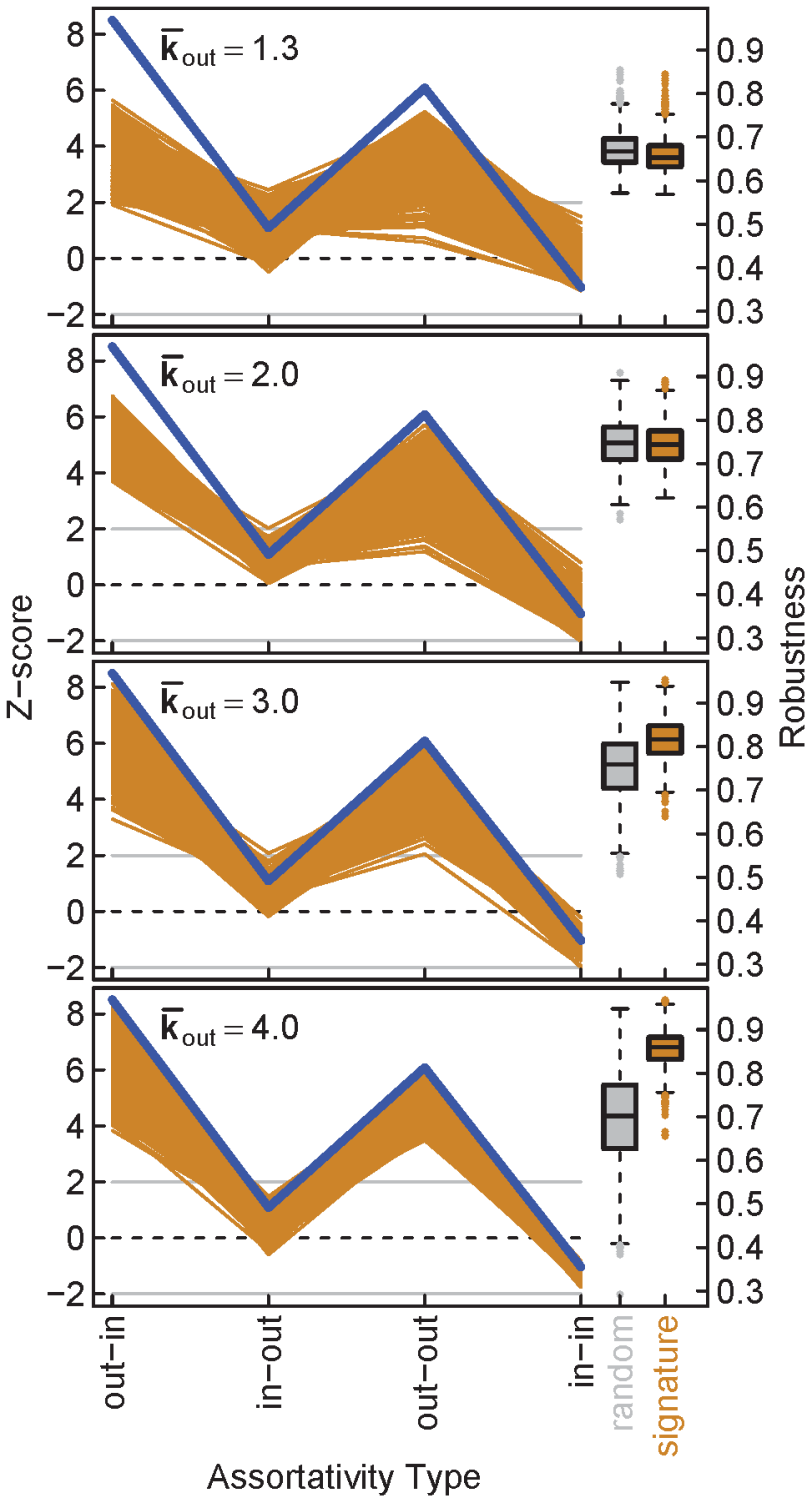
Dense TFN models that possess the human assortativity signature are more robust than random models. Z-scores for the four types of assortativity are represented as signatures, as in Fig. 2. The average human assortativity signature was computed from the signatures of the 41 human TFNs, and is represented as a blue line. For each average out-degree 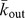, 1000 TFN models (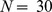) were generated to approximate the human signature, and the resulting signatures are shown as orange lines. For each TFN model, we constructed 1000 randomly-rewired null models for computing Z-scores. Box-and-whisker plots show the robustness for the 1000 TFN models that approximate the human signature (orange) compared to 1000 random models (grey). For 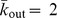, 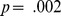, and for all other 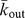, 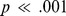 (paired *t*-test).

In this fashion, we compared TFN models that closely resembled the human TFNs to random TFN models that were constructed without considering assortativity or average IC size. For less dense TFN models, we found that the human signature did not confer robustness compared to random models (Fig. 3, average out-degree 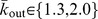). In contrast, dense TFN models with the human signature displayed marked increases in robustness over random models (Fig. 3, 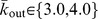). Specifically, the average robustness increased by 9% and 25%, respectively. Considering the increased out-out assortativity in the human signature, each of these observations is consistent with previous work which showed that the robustness of TFN models is not closely related to out-out assortativity when 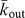 is small, but is positively correlated with out-out assortativity when 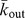 is large [21], [22]. Given the large 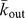 of the 41 human TFNs (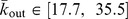), this suggests that the increased out-out assortativity in the human signature contributes to increased robustness. However, since the previously established link between out-out assortativity and robustness does not take into account the three other types of assortativity, these components of the human signature must be evaluated explicitly for their respective influence on robustness.

### Out-Out Assortativity Is the Main Driver of Robustness

**Figure 4:**
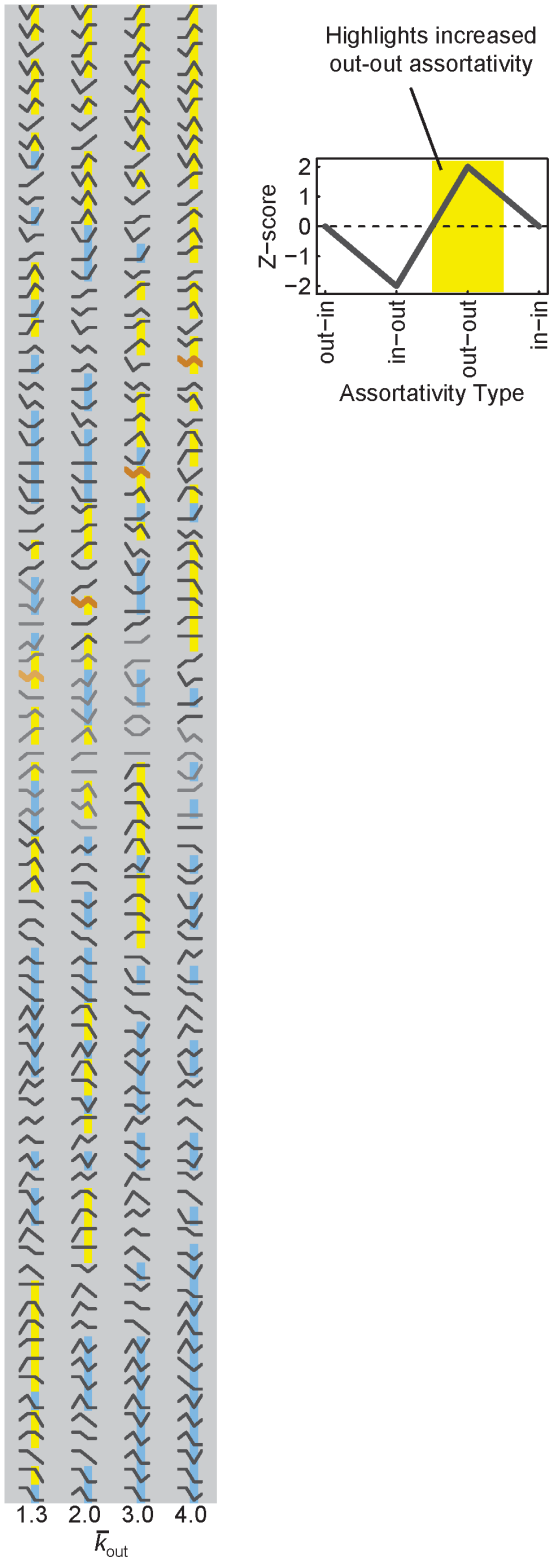
TFN models incorporating 81 different assortativity signatures highlight out-out assortativity as driving the robustness of dense TFNs. Each assortativity signature contains a different combination of the four types of assortativity where 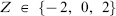 (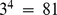). We built 1000 TFN models for each signature, and measured their robustness. Signatures in each column are sorted top-to-bottom in decreasing order by the average robustness of the 1000 TFN models. Faded signatures are not significantly different from the average robustness of random TFN models (paired *t*-test; significant Bonferroni-corrected 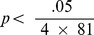). Yellow highlights signatures where 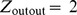 and blue highlights signatures where 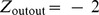. The orange lines correspond to the signature that is most similar to the average human signature (Fig. 3).

In order to address the question of how the various components of the human signature influence robustness, we created TFN models that approximate 81 different signatures. These signatures were selected based on all possible combinations of less-than-expected (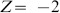), expected (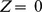), and greater-than-expected (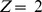) Z-score values for each of the four components of an assortativity signature (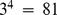; see Methods). For each 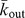, the signatures were ranked by the average robustness of their TFN models, and statistically compared to random TFN models (Fig. 4).

Of the 81 signatures, the one that most closely resembles the human signature consists of greater-than-expected out-in and out-out assortativity, along with expected in-out and in-in assortativity (Fig. 4, orange lines). For small 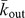, this signature displays random or near-random robustness (Fig. 4, 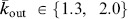), whereas for large 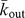, this signature displays increasing robustness (Fig. 4, 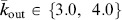). This is evident in the robustness rank of this signature, which rises from 37^th^ to 20^th^ (out of 81) as 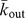 increases. This is consistent with the observation that the human signature becomes increasingly robust compared to random TFN models as 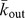 increases (Fig. 3). To qualitatively inspect whether increased out-out assortativity plays a role in the robustness rankings of the 81 signatures, Fig. 4 displays the signatures ordered by their average robustness and highlights those with greater-than- or less-than-expected out-out assortativity in yellow or blue, respectively. As 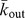 increases, the separation between yellow- and blue-highlighted signatures becomes more pronounced, with yellow occupying many of the top and blue occupying many of the bottom rankings. This hints that as 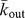 increases, out-out assortativity becomes more influential in determining robustness.

**Figure 5:**
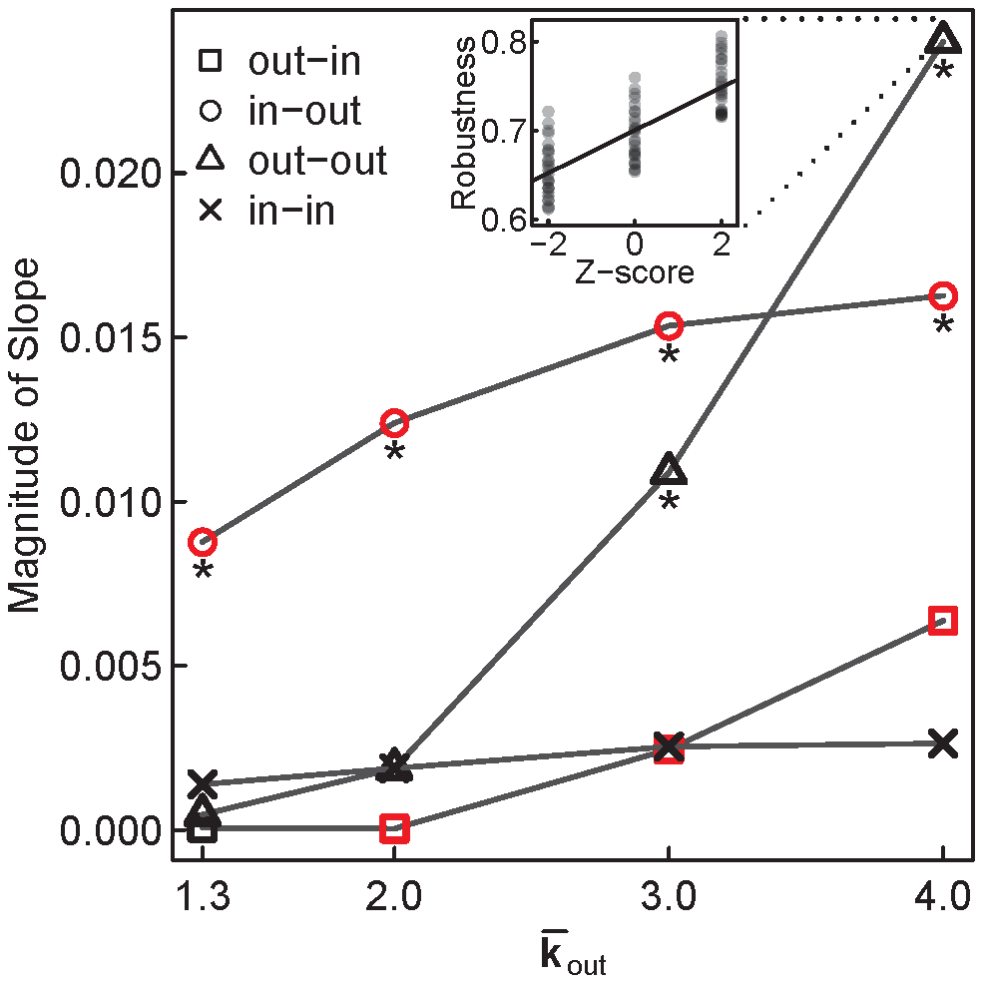
Of the four components of the assortativity signature, out-out assortativity is the strongest predictor of robustness in dense TFN models. Simple linear regression was used to explain the variation in the average robustness for the 81 test signatures (as shown in Fig. 4). For each 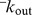, the Z-score for each assortativity type was used as the lone explanatory variable, resulting in a total of 16 linear models. Black points represent positive slopes of best fit lines (*e.g.*, see inset), and red points represent negative slopes. Slopes are significant (asterisks) if 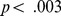 (Bonferroni-corrected, 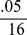).

To quantitatively assess how much influence each of the four components of the assortativity signature exerts over robustness, we employed simple linear regression. For each combination of 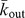 and assortativity type, the independent variable was the Z-score of that assortativity type (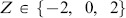), and the dependent variable was the average robustness of the signature (Fig. 5). The slopes of these linear models reveal to what extent each component of the signature affects robustness. For all 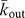, in-out assortativity maintains a strong negative influence over robustness, and for small 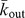, it has the strongest effect on robustness (Fig. 5, circles). However, as 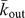 increases, out-out assortativity has an increasingly strong positive influence over robustness, and for 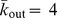 it is the component that exerts the strongest influence (Fig. 5, triangles). Thus for dense TFNs, out-out assortativity is the component of the signature that contributes the most to robustness. In the case of the human signature, in-out assortativity does not significantly differ from random in 34 of 41 human TFNs (Fig. 2), and is unlikely to exert a strong negative influence on robustness. This leaves the increased out-out assortativity in 40 of 41 human TFNs (Fig. 2) as the key component governing the increase in the robustness of dense TFN models that approximate the human signature (Fig. 3).

## Discussion

We have used DNaseI-seq data [13] to characterize the assortativity signatures of human transcription factor networks (TFNs) with between 485 and 526 sequence-specific transcription factors, revealing a common assortativity signature amongst 41 distinct cell and tissue types. This signature consists of greater-than-expected values for both out-in and out-out assortativity, along with values for in-out and in-in assortativity that do not differ from the null expectation. Perturbation analyses of TFN models demonstrated that the assortativity signature has a pronounced influence on the robustness of a TFN's gene expression phenotypes. Moreover, out-out assortativity is the most important of the four components of the assortativity signature in driving this robustness in TFN models that begin to approach the high edge density of the human TFNs. This is consistent with earlier theoretical results that showed the relationship between this type of assortativity and robustness [21], [23].

Experimental work has repeatedly demonstrated the robustness of TFNs to various forms of perturbation [28], [29], including noisy gene expression [30], gene knockouts [31], and the rewiring of regulatory interactions [32]. The robustness of biological networks stems from several structural sources, ranging from their heavy-tailed degree distributions [33] to their overrepresentation of autoregulatory subgraphs [34]. The results presented here suggest that degree assortativity provides an additional structural source of robustness in biological networks, and that human TFNs share an assortativity signature that confers such robustness.

The observation that the human assortativity signature displays differences among the four types of assortativity is broadly consistent with previous work, which has shown that real-world directed networks are rarely entirely assortative or disassortative [24]. Indeed, barring a strong correlation between the in- and out-degrees of a network (the human TFNs show only weak correlations, Pearson's 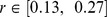), a neutral or adaptive network rewiring process would be capable of modifying one component of the signature without dramatically altering another. Such rewiring is easily achieved by mutations in *cis*-regulatory regions, such as point mutations or indels, that are capable of adding or eliminating regulatory interactions between a TF and its target genes [35], and evidence from comparative genomics shows that this is common in the evolution of both microbes [36], [37] and vertebrates [38], [39].

Genomic footprinting is not the only method that has been used to incorporate sets of human TF-DNA binding events into TFNs. For example, chromatin immunoprecipitation of individual TFs combined with high-throughput sequencing (ChIP-seq) has also been used for examining human TFN topology [12], and this approach has the advantage of generating direct TF-DNA binding data without the need to infer TF identity. However, the extent to which ChIP-seq data can be used to construct genome-wide TFNs is limited by (1) the availability of high affinity antibodies for individual TFs, and (2) the need to perform deep sequencing separately for each TF in each cell line. Recently, combining the data from hundreds of ChIP-seq experiments across multiple cell lines resulted in a human TFN containing 119 TFs [12], but this massive dataset still represents only a small fraction of the approximately 1400 TFs encoded in the human genome [40]. Thus, for the purposes of exploring global topological properties of human TFNs, genomic footprinting provides a few important advantages. First, the TFNs are substantially larger than those that can currently be obtained using ChIP-seq data (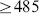 vs. 119 TFs). Although the human TFNs obtained from DNaseI-seq are large and densely connected, the estimated false discovery rate (FDR) of TF-DNA binding events is quite low (1%; [25]), and a sensitivity analysis suggests that this level of false-positive binding does not produce any substantial change to the assortativity signatures of these TFNs (Fig. S1). Second, it is not necessary to combine data from multiple cell lines in order to generate large TFNs. This last point is crucial, as it frees us from the assumption that the topology of a combined TFN approximates topologies realized by individual cell types. Notably, this assumption appears to be unwarranted for these TFNs, as their union displays a markedly different topology from the individual TFNs [13].

Characterizing the regulatory networks that govern the development, physiology, and behavior of organisms is a central goal of modern genomics [3], [11]. One of its main challenges is the interpretation and synthesis of the wealth of data generated by the various high-throughput technologies used in this endeavor, a challenge that stems in part from the wide variety of post-processing techniques associated with each technology. For example, the topological properties of the TFN constructed using ChIP-seq [12] depend heavily upon the post-processing techniques used for peak calling and target gene assignment, as these choices impact the set of DNA sequences considered bound by a transcription factor [41] and the regulatory interactions included in the TFN [42]. When target genes are assigned using a peak calling algorithm coupled with a window-based approach (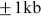 of the transcription start site), the assortativity signature of the TFN is qualitatively similar to that observed using DNaseI-seq data (Fig. S2). In contrast, when target genes are assigned using a probabalistic model of TF binding (TIP) that implicitly takes peak intensity and distance from the transcription start site into account [43], the assortativity signature of the TFN differs substantially from that observed using DNaseI-seq data (Fig. S2). Such discrepancies are problematic, because it is difficult to ascertain which TFN best represents the true regulatory network, and they highlight the importance of understanding whether and how different technologies and data post-processing techniques bias our understanding of TFN topologies.

One of the advantages of constructing TFNs from the DNaseI-seq data of Neph et al. [13] is that a common post-processing pipeline was used for each of the 41 diverse cell and tissue types, allowing for a direct comparison of the assortativity signatures of these TFNs. It is striking that regardless of tissue origin, transformation, or differentiated state, all TFNs possessed remarkably similar assortativity signatures. This parallels the common network architecture observed through the analysis of three-node subgraphs in these networks [13]. The absence of markedly different signatures might suggest a core topology that is shared across different cell types, and that functionally driven cell-type specific network rewiring [13] ultimately converges on that core topology. Alternatively, the shared topology could reflect that this dataset captures proximal regulatory interactions while ignoring those that are distal. Epigenetic marks, such as histone methylation, show large variations between cell types at distal enhancer sites, indicating that transcription factor binding is more cell-type specific at enhancers than at promoters [44]. Understanding how the inclusion of such distal regulatory information might affect the assortativity signatures of diverse cell and tissue types, and how this in turn may affect the robustness of the resulting TFNs, presents an exciting direction for future research.

Another advantage of this dataset is its size. Comprising genome-wide binding information for between 485 and 526 transcription factors, this dataset is considerably larger than any other used for constructing human TFNs [12], [13]. Nevertheless, it comprises only an approximate third of all human transcription factors [40]. It is therefore important to understand how the assortativity signatures of the TFNs constructed here may be affected by the number of transcription factors in the dataset. To this end, we performed a sensitivity analysis in which we randomly removed a proportion of the transcription factors from the dataset, constructed the resulting transcription factor subnetwork, and analyzed its assortativity signature. Fig. S3 shows that the reported assortativity signature is insensitive to the removal of up to 60% of the transcription factors for a stromal cell type. Similar insensitivities were observed across all 41 cell and tissue types. This is consistent with a feature that was observed during the initial analysis of these TFNs. Specifically, Neph et al. [13] removed 63 TFs from their analysis, as each of those TFs possessed overlapping or duplicate DNA-binding motifs that could not be distinguished from another TF that was ultimately included in the TFNs. In doing so, they found that this did not substantially affect the architecture of the TFNs as characterized by the frequency of three-node subgraphs. This may indicate that as the number of known TF-binding motifs grows, and the number of similar or overlapping motifs grows, the topology of the TFNs will remain relatively stable. To test this hypothesis, it will be necessary to incorporate the growing body of TF-binding motif data made available through high-throughput methods, such as protein-binding microarrays [45] and HT-SELEX [46].

In addition to evaluating the sensitivity of TFN topology to random TF removal, we also sought to understand what happens to the assortativity signature upon removal of the most highly connected TFs, referred to here as hubs. To this end, we incrementally removed the hub TFs and determined the assortativity signatures of the resulting networks (see Methods). Signatures were relatively sensitive to this procedure, changing markedly upon the removal of the top 5% of hub TFs (Figs. S4, S5). These changes tended to take one of several forms. In some cases, only one component of the signature was sensitive to the removal of hub TFs (Fig. S4, left), whereas in other cases, multiple components were sensitive (Fig. S4, right). Out-out assortativity, the component that emerged as the most important to the robustness of dense TFN models (Fig. 5), likewise displayed variation in its sensitivity to hub TF removal (Fig. S4). These results suggest that the assortativity signatures of currently established human TFNs will be prone to changes if additional, highly connected TFs are included. However, this analysis also suggests that out-out assortativity is at least partially insensitive to even these drastic changes to network topology. For example, whereas the out-in assortativity of nearly all of the TFNs (38 of 41) was sensitive to the removal of the top 2% of the hub TFs, the out-out assortativity of only a third of the TFNs (14 of 41) was similarly sensitive (Fig. S5).

Computational models of TFNs are commonly used to study the spatiotemporal dynamics of transcriptional regulation [47]–[49] and the sensitivity of these dynamics to environmental [19], [50], [51] and genetic perturbation [52], [53]. To do so accurately, the structure of TFN models are often engineered to reflect one or more salient topological properties of known regulatory networks. For example, the out-degree distribution is often chosen from a suite of heavy-tailed distributions, reflecting a statistical feature of organisms as different as microbes [52] and humans [12]. Similarly, TFN models have been engineered to possess a modular structure [17], which is considered a fundamental characteristic of biological regulatory networks [54]. Our findings suggest that in addition to these topological properties, it will be informative to consider the important components of the assortativity signature in any work designed to advance the theoretical understanding of networked systems.

## Methods

### Assortativity

The assortativity of an undirected network measures the extent to which the nodes at both ends of an edge have similar degrees (numbers of connections). This is computed as the Pearson correlation coefficient of the degrees of all pairs of nodes that have an edge between them [20]: 
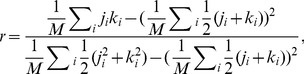
(1)where 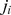 and 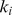 are the degrees of the nodes separated by the 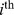 edge, and 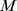 is the number of edges in the network.

In a directed network, such as a TFN, each node possesses both an in-degree and an out-degree, defined as the number of incoming and outgoing connections (respectively) for that node. There are thus four types of assortativity, one for each of the four possible combinations of in- and out-degree: out-in, in-out, out-out, and in-in assortativity. These were calculated as follows [24]: 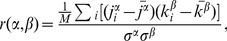
(2)where 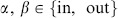, 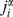 is the 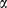-degree of the source node of the 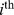 edge, 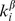 is the 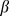-degree of the target node of the 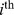 edge, 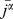 and 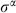 are the mean and standard deviation of the 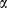-degree of the source nodes for all edges (
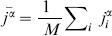
, 
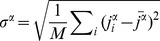
), likewise for the mean and standard deviation of the 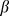-degree of the target nodes for all edges (
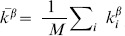
, 
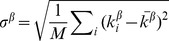
), and 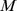 is the number of edges in the network.

### In-components and Characteristic Path Length

The in-component (IC) of a node 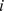 is the set of nodes from which there exists a directed path to node 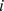 [55]. In other words, in a TFN, the IC of a TF 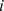 is the set of TFs that either lie upstream of 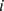 in its regulatory pathway or provide feedback to 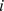. The size of the IC of 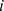 is thus the number of nodes in this set (including 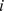 itself), and the average IC size was calculated simply as the mean of the IC sizes for all nodes in the network.

The characteristic path length (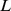) of a directed network is the average length of the shortest directed path between any two nodes 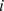 and 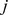. In a TFN, it is the average number of regulatory links between two TFs. The shortest paths were determined using a breadth-first search algorithm.

### Random Networks and Z-Scores

Random networks were generated for each human TFN using an edge-swapping algorithm that preserves the in- and out-degree of every node while randomizing which pairs of nodes are connected [56]. This preservation of degree distribution is essential, both because a degree distribution has a pronounced influence on network dynamics [57]–[59], including those of model regulatory networks [18], [60], and because the expected assortativity signature varies among networks with different degree distributions [61]. By holding the degree distribution fixed for each human TFN, the resulting random networks can be used to interrogate whether assortativity deviates from what is expected at random given the observed degree distribution. A single iteration of this algorithm first considers two edges 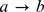 and 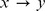. Swapping these edges produces 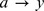 and 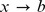. If these two edges do not already exist in the network, then the new edges remain and the old edges are discarded. Beginning with a human TFN, 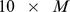 edge-swaps were performed, where 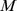 is the number of edges in the TFN. This resulted a single random network. The process was repeated to generate 1000 random networks for each human TFN.

Self-loops were removed from the human TFNs prior to random network generation, and were subsequently prevented from reoccurring in random networks. This was done because the presence of self-loops trivially inflates all four assortativity values. Through their removal, assortativity can be examined separately from any potential enrichment for self-loops. This results in a more conservative estimate of how assortativity differs from the null expectation.

Z-scores were used to enable the direct comparison of the human TFNs with respect to assortativity and 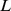. The Z-score of a value reflects its distance from its expectation under the assumption that the values are normally distributed, and its use here thus depends on the assumption that the random networks generated for each TFN possess normally distributed network properties. This assumption was supported (*i.e.*, the null hypothesis of normality was not rejected) for all of the TFNs for out-in, in-out, out-out, in-in assortativity, and 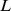 (Kolmogorov-Smirnov test, 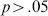). The Z-scores for each of these properties were calculated separately for each human TFN, as follows. First, the null distribution for a particular topological property of the human TFN was calculated from the 1000 random networks (described above). The Z-score was then calculated as 
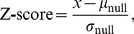
(3)where 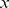 is the observed value of the topological property, 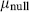 is the mean of the null distribution, and 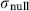 is the standard deviation of the null distribution. A Z-score of less than -2 or greater than 2 was used to assign statistical significance. These thresholds follow a similar analysis [24], and represent a confidence level of approximately 95% for each individual test. Together, the Z-scores for the four types of assortativity (out-in, in-out, out-out, and in-in) formed the assortativity signature of the TFN.

In contrast to assortativity and 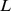, mean IC sizes were not normally distributed among the random networks for any human TFN (Kolmogorov-Smirnov test, 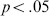). Therefore, instead of computing a Z-score, the mean IC size of a TFN was determined to differ significantly from its null expectation if its value lay outside the middle 95% of the null distribution.

### TFN Models

Transcription factor networks (TFNs) were modeled as random Boolean networks [26], where nodes represent TFs and edges represent regulatory interactions between TFs (Fig. 1B). The dynamics of these TFN models produce simulated gene expression patterns, as follows. At a discrete time 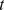, each node 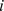 possesses a Boolean state 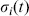 that encodes whether or not 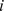 is present as protein at time 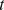. The state of 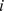 at the next time point is updated according to a deterministic Boolean function that takes as inputs the present states of the regulators of 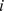: 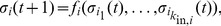
(4)where 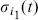 is the state of the first regulator, and there are 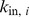 regulators for node 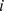. Each node has its own Boolean function, and together they form the set of regulatory rules, which we consider to be the genotype of the TFN model (Fig. 1C). The set of states for all nodes at time 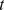 is referred to as the configuration at that time, and given an initial configuration, the regulatory rules synchronously update the configuration to the next time point. Updating the configuration proceeds until a configuration is reached that exactly matches one of the configurations encountered previously (Fig. 1D). This is guaranteed to occur as there are a finite number of possible configurations (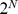, where 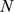 is the number of nodes). Because the regulatory rules update configurations synchronously and deterministically, subsequent updates will eventually reproduce the same configuration(s) seen before, resulting in a steady-state attractor. The attractor represents a stable gene expression pattern produced by the TFN model, and is thus regarded as its phenotype.

Random Boolean networks are both general and abstract, making them a useful tool for studying the genotype-to-phenotype relationship in genetic regulation. They also make a number of simplifying assumptions. For example, these models assume that gene expression is Boolean, when in reality mRNA and protein concentrations are quantitative traits. Even under such an assumption, random Boolean networks have accurately recapitulated the dynamics of a number of model experimental systems. For example, they have been used to model the spatiotemporal gene expression patterns in the developing sea urchin embryo [49], the circadian oscillations of gene expression in both the fungus *Neurospora crassa* and the plant *Arabidopsis thaliana* [62], and the p53-dependent fate of a human breast cancer cell line exposed to a therapeutic agent [63].

In another simplifying assumption, these models synchronously update the states of all nodes at each time point, whereas in real biological systems genetic regulation is asynchronous. Although relaxing this assumption can lead to differences in attractors [64], the methods employed in this present study do not rely specifically on attractor identity, but instead depend on how easily ensembles of attractors are perturbed (see Robustness, below). Furthermore, the computational feasibility of this study would be compromised by trying to account for the large number of asynchronous update orderings.

### Generating TFN Models with Assortativity Signatures

Weakly connected TFN models without self-loops were used to approximate the human signature and the set of 81 different assortativity signatures. Self-loops were excluded to match their removal from the human TFNs (see Random Networks and Z-scores), and this has been shown to not significantly alter the dynamics of these models [22]. TFN models were constructed by randomly connecting 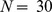 nodes using a power-law degree distribution, which is thought to better approximate real-world TFNs than alternative distributions [52]. For each TF, the probability of selecting 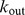 targets depended on the exponent 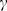: 
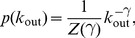
(5)where 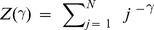. TFN models with different edge densities were constructed using 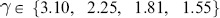, resulting in an average number of targets 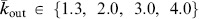. Since the dynamics of random Boolean networks are heavily influenced by their dynamical regime, these 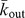 values were selected such that all three dynamical regimes were represented. Here, TFN models with 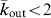 possess ordered dynamics, 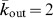 possess critical dynamics, and 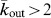 possess chaotic dynamics. Increasingly dense TFN models are computationally difficult to simulate, as they generate increasingly complex phenotypes (long attractors), and thus extensive simulations of 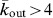 were not computationally feasible. For each 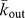, 1000 random TFN models were generated.

Each of these random TFN models was then rewired to generate new TFN models (weakly connected and without self-loops) that approximated specific assortativity signatures, as follows. For each random TFN model, edge-swapping was used to build a null distribution of networks (see Random Networks and Z-scores). This enabled the conversion between raw assortativity values and Z-scores for that model. Then, the random TFN model was rewired as described previously, however in this case new edges were only kept if the resulting change in network topology either maintained or decreased the Euclidian distance between the four assortativity Z-scores of the network and those of the desired signature. Rewiring concluded either upon achieving the signature to within a distance of 0.0001 or after 10000 edge-swaps that failed to make progress toward the signature. Additionally, during rewiring, the mean IC size of the model was restricted by precluding edge-swaps that would have increased or decreased this value beyond the middle 20% of the null distribution for that model. This more accurately reflects the fact that most of the human TFNs possess mean IC sizes that are not significantly different from expected (Fig. 2). Rewiring of the random TFN models resulted in 1000 TFN models for each signature, where the precise combination of in- and out-degrees present in each of the random TFN models was also represented for each signature.

### Robustness

The phenotypic robustness of a TFN model was estimated by computing a random walk through the space of genotypes that produce the same phenotype, as described previously [21]. This random walk was conducted as follows. A genotype (regulatory rules, Fig. 1B) for the TFN model was constructed at random, such that there was an equal probability of choosing either a 0 or a 1. Then, a random initial configuration was used to generate a phenotype (attractor). A step in the random walk was attempted by flipping one of the bits of the genotype, and regenerating the phenotype using the same initial configuration as before. If the original phenotype was recovered, then the step was successful and the mutated genotype was kept. Otherwise, the mutation was reverted to yield the previous genotype. Note that during this process, network topology (as defined by TF-TF edges) is not altered by mutations, and it is strictly the genotype (regulatory rules) that is mutated. This process was repeated for 500 attempted steps, and the proportion of successful steps served as an estimate of the robustness for that particular phenotype. One random walk was performed for each of 100 different combinations of random genotypes and initial configurations, and the resulting proportions were averaged to produce an estimate of the phenotypic robustness for the TFN model.

### Sensitivity Analysis

The sensitivity of each of the 41 human TFNs to random node removal was performed by randomly removing 20%, 40%, or 60% of the total nodes in the network. For each of these values, 100 subnetworks were generated by removing random sets of nodes, and for each of these subnetworks a null distribution of 100 networks was generated by performing edge-swaps, as described previously. This enabled the conversion of assortativity values into Z-scores, and the average of the Z-scores for the 100 subnetworks served as an approximation of the assortativity signature for that level of node removal.

The sensitivity of the human TFNs to hub TF removal was performed by removing the top 1%, 2%, 3%, 4%, or 5% of hubs, as determined by the total degree (sum of in- and out-degrees) of each TF. Hub removal was only performed once for each TF and each level of hub removal, since hubs were chosen for removal in a deterministic fashion. The signature for each resulting subnetwork was computed as described above. The signatures for the subnetworks were then used to determine the sensitivity of each component of the assortativity signature for each TFN. For each level of hub TF removal, a particular component of the assortativity signature of a TFN was determined to be sensitive if that component of the new signature possessed a different relationship to its null expectation than observed in the original signature. For example, if out-out assortativity was greater-than-expected in the original TFN but did not differ from the null expectation in the new subnetwork, then out-out assortativity in that TFN was determined to be sensitive to that level of TF hub removal. On the other hand, if the new signature instead showed greater-than-expected out-out assortativity, then that component of the signature of the TFN was not sensitive to that level of TF hub removal.

The sensitivity of human TFNs to TF-TF edge removal was performed by randomly removing 0.5%, 1%, or 2% of the total edges in the network. For each of these values, 100 subnetworks were generated by removing random sets of edges, and Z-scores were calculated as described above.

## Supporting Information

Figure S1 **The assortativity signature for a stromal cell type (AG10803) is insensitive to edge removal.** Varying proportions (0.5%, 1%, or 2%) of TF-TF edges were removed from the AG10803 TFN, and average assortativity signatures were calculated for the subnetworks (see Methods). The original signature is displayed, along with the 95% confidence intervals for the subnetwork signatures. This particular stromal cell type (AG10803; Table S1) is shown as a representative example.
(TIFF)

Figure S2 **TFN assortativity signatures are sensitive to the method used for identifying TF targets.** The average human signature derived from the DNaseI-seq TFNs and presented in this paper (blue) is shown with the two signatures for the proximal TFNs assembled from ChIP-seq data [12]. One of these ChIP-seq TFNs was derived by using a peak-calling algorithm and window-based gene assignment on the ChIP-seq data (solid brown), and the other TFN by using a window-free probabilistic model of TF binding (TIP; dashed brown) on the same data. These two TFNs were downloaded from http://encodenets.gersteinlab.org/, where they are labeled as “raw” (window-based) and “filtered” (TIP). All self-loops were removed. Signatures were calculated as described in Methods.
(TIFF)

Figure S3 **The assortativity signature for a stromal cell type (AG10803) is insensitive to node removal.** Varying proportions (20%, 40%, or 60%) of nodes were removed from the AG10803 TFN, and average assortativity signatures were calculated for the subnetworks (see Methods). The original signature is displayed, along with the 95% confidence intervals for the subnetwork signatures. This particular stromal cell type (AG10803; Table S1) is shown as a representative example.
(TIFF)

Figure S4 **The sensitivity of the assortativity signature to hub TF removal depends on the TFN.** Varying proportions (1–5%) of hub TFs, defined as the most highly connected TFs according to the sum of their in- and out-degrees, were removed from each of the 41 human TFNs, and the new assortativity signature in each case was calculated (see Methods). The signatures for stromal and visceral cell types are shown as representative examples of TFNs where the signature is perturbed by hub TF removal. The original signatures are displayed as black lines, and shaded lines represent the signatures after hub TF removal (see legends). This particular stromal cell type (AG10803; Table S1) is shown as representative example of TFNs where much of the signature was relatively insensitive to hub TF removal. In contrast, the visceral cell type (HA-h; Table S1) is shown as a representative example of TFNs where the signature was heavily perturbed by hub removal.
(TIFF)

Figure S5 **There is variation in the sensitivity of the four assortativity signature components to hub TF removal.** Varying proportions (1–5%) of hub TFs, defined as the most highly connected TFs according to the sum of their in- and out-degrees, were removed from each of the 41 human TFNs, and the assortativity signature in each case was calculated (see Methods). For each proportion, the y-axis displays separately for each type of assortativity the number of TFNs that were sensitive to that level of hub TF removal (see Methods).
(TIFF)

Table S1 **Human transcription factor networks.** Networks were downloaded from www.regulatorynetworks.org (v09042012) [13], and self-loops were removed.
(PDF)
