# Supplementary figures and images for: Phenotypic Robustness and the Assortativity Signature of Human Transcription Factor Networks
Source: PLoS Comput Biol. 2014 Aug 14;10(8):e1003780. doi: 10.1371/journal.pcbi.1003780 (PMC4133045; doi:10.1371/journal.pcbi.1003780)

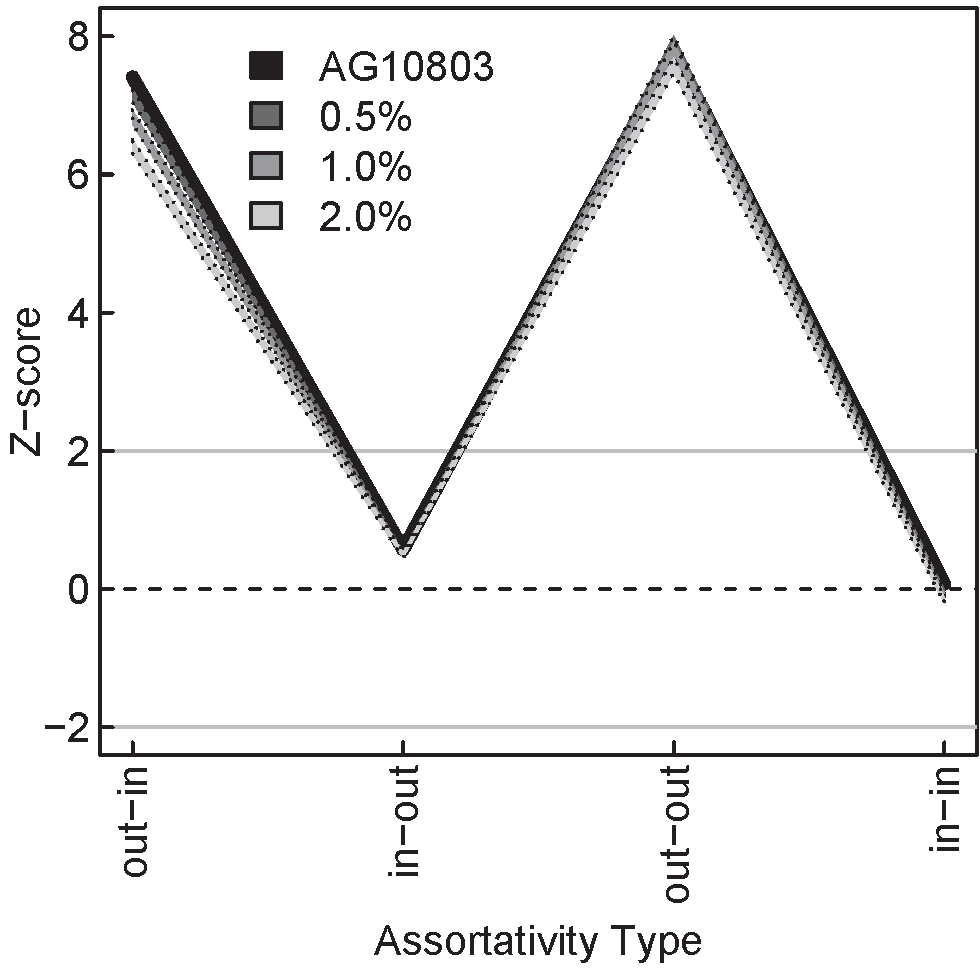

Supplement: Figure S1 — The assortativity signature for a stromal cell type (AG10803) is insensitive to edge removal. Varying proportions (0.5%, 1%, or 2%) of TF-TF edges were removed from the AG10803 TFN, and average assortativity signatures were calculated for the subnetworks (see Methods). The original signature is displayed, along with the 95% confidence intervals for the subnetwork signatures. This particular stromal cell type (AG10803; Table S1) is shown as a representative example. (TIFF) [file pcbi.1003780.s001.tiff]

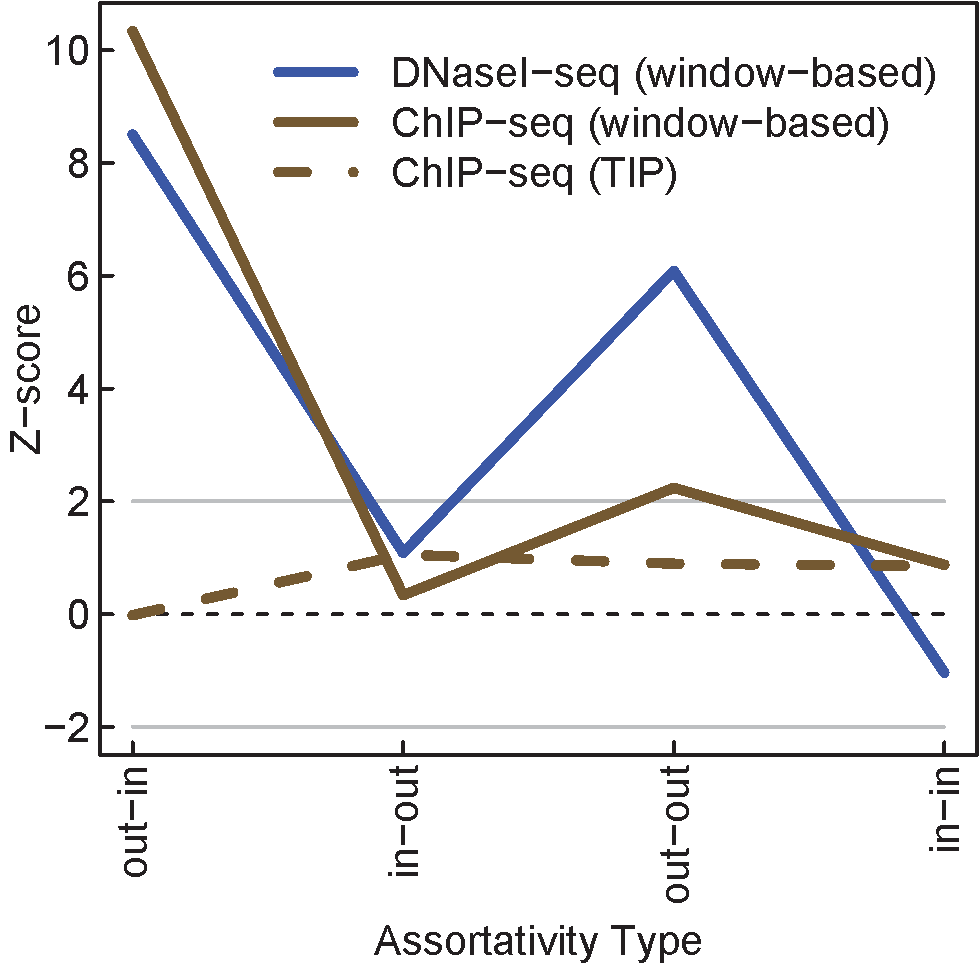

Supplement: Figure S2 — TFN assortativity signatures are sensitive to the method used for identifying TF targets. The average human signature derived from the DNaseI-seq TFNs and presented in this paper (blue) is shown with the two signatures for the proximal TFNs assembled from ChIP-seq data [12]. One of these ChIP-seq TFNs was derived by using a peak-calling algorithm and window-based gene assignment on the ChIP-seq data (solid brown), and the other TFN by using a window-free probabilistic model of TF binding (TIP; dashed brown) on the same data. These two TFNs were downloaded from http://encodenets.gersteinlab.org/, where they are labeled as “raw” (window-based) and “filtered” (TIP). All self-loops were removed. Signatures were calculated as described in Methods. (TIFF) [file pcbi.1003780.s002.tiff]

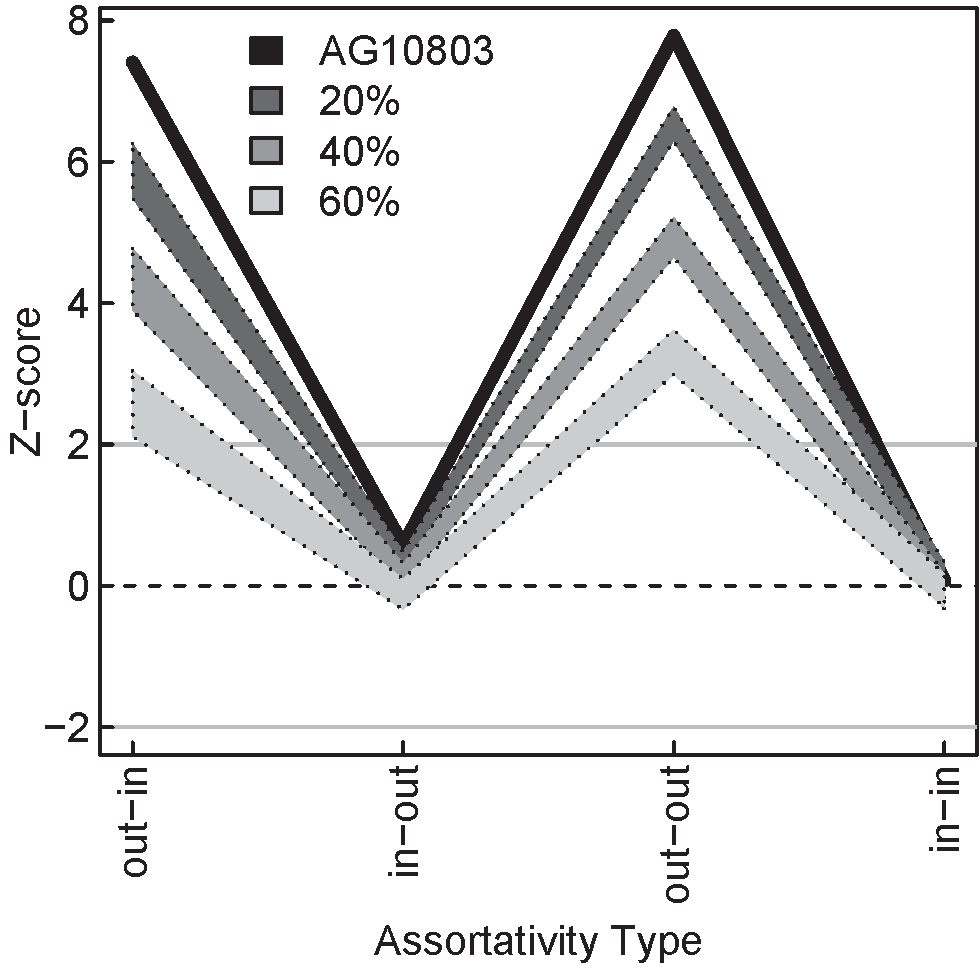

Supplement: Figure S3 — The assortativity signature for a stromal cell type (AG10803) is insensitive to node removal. Varying proportions (20%, 40%, or 60%) of nodes were removed from the AG10803 TFN, and average assortativity signatures were calculated for the subnetworks (see Methods). The original signature is displayed, along with the 95% confidence intervals for the subnetwork signatures. This particular stromal cell type (AG10803; Table S1) is shown as a representative example. (TIFF) [file pcbi.1003780.s003.tiff]

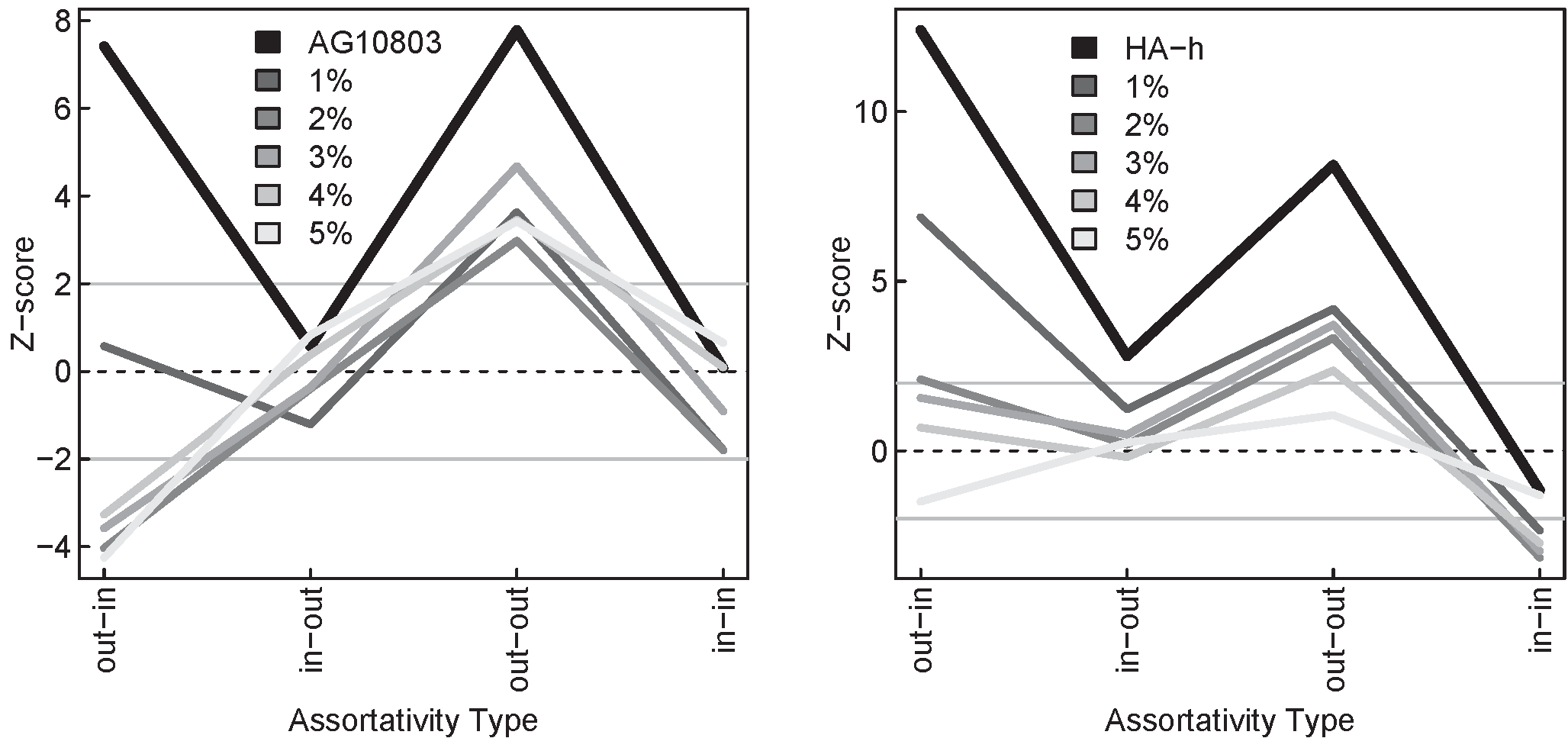

Supplement: Figure S4 — The sensitivity of the assortativity signature to hub TF removal depends on the TFN. Varying proportions (1–5%) of hub TFs, defined as the most highly connected TFs according to the sum of their in- and out-degrees, were removed from each of the 41 human TFNs, and the new assortativity signature in each case was calculated (see Methods). The signatures for stromal and visceral cell types are shown as representative examples of TFNs where the signature is perturbed by hub TF removal. The original signatures are displayed as black lines, and shaded lines represent the signatures after hub TF removal (see legends). This particular stromal cell type (AG10803; Table S1) is shown as representative example of TFNs where much of the signature was relatively insensitive to hub TF removal. In contrast, the visceral cell type (HA-h; Table S1) is shown as a representative example of TFNs where the signature was heavily perturbed by hub removal. (TIFF) [file pcbi.1003780.s004.tiff]

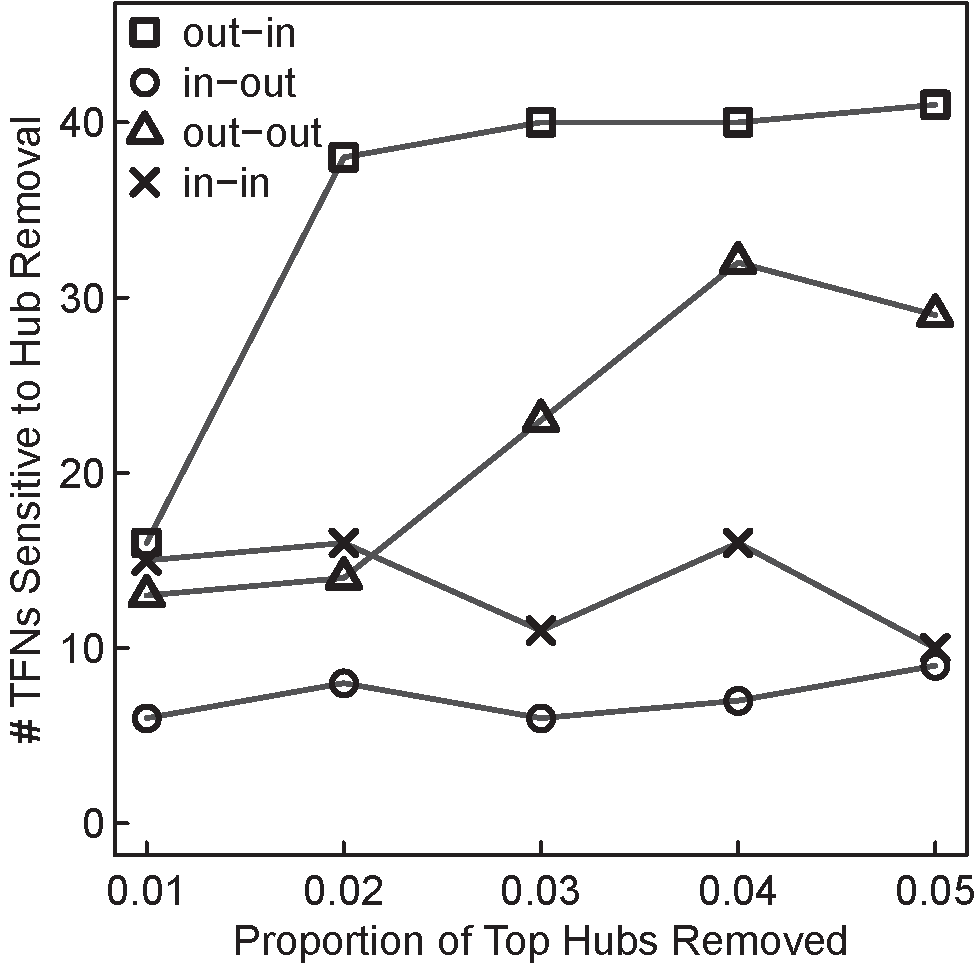

Supplement: Figure S5 — There is variation in the sensitivity of the four assortativity signature components to hub TF removal. Varying proportions (1–5%) of hub TFs, defined as the most highly connected TFs according to the sum of their in- and out-degrees, were removed from each of the 41 human TFNs, and the assortativity signature in each case was calculated (see Methods). For each proportion, the y-axis displays separately for each type of assortativity the number of TFNs that were sensitive to that level of hub TF removal (see Methods). (TIFF) [file pcbi.1003780.s005.tiff]
