# Supplementary material for: Phenotypic Robustness and the Assortativity Signature of Human Transcription Factor Networks
Source: PLoS Comput Biol. 2014 Aug 14;10(8):e1003780. doi: 10.1371/journal.pcbi.1003780 (PMC4133045; doi:10.1371/journal.pcbi.1003780)
Supplement: Table S1 — Human transcription factor networks. Networks were downloaded from www.regulatorynetworks.org (v09042012) [13], and self-loops were removed. (PDF) [file pcbi.1003780.s006.pdf]

**Cell Line**	**Cell Type**	**Tissue Type**	**No. TFs**	**No. Edges**
HRCEpiC	Renal Cortical Epi.	Epithelia	518	9412
HCPEpiC	Choroid Plexus Epi.	Epithelia	520	13570
SAEC	Small Airway Epi.	Epithelia	512	9669
HAEpiC	Amnioitic Epi.	Epithelia	518	13031
HEEpiC	Esophageal Epi.	Epithelia	520	14192
HIPEpiC	Iris Pigment Epi.	Epithelia	520	12203
CD34+	Hemat. Stem Cell	Blood	519	16005
NB4	Promyelocytic Leuk.	Blood	517	18348
K562	Erythroid	Blood	485	8821
Th1	T-Lymphocyte	Blood	510	12541
CD20+	B-Lymphocyte	Blood	507	16315
GM06990	B-Lymphoblastoid	Blood	496	12742
GM12865	B-Lymphoblastoid	Blood	505	14730
HMVEC_dBlAd	Adult Dermal Blood	Endothelia	515	13236
HMVEC_dBlNeo	Neonatal Dermal Blood	Endothelia	520	16425
HMVEC_LLy	Lung Lymphatic	Endothelia	515	15047
HMVEC_dLyNeo	Neonatal Dermal Lymph.	Endothelia	519	15193
fBrain	Fetal Brain	Fetal tissues	513	11459
fHeart	Fetal Heart	Fetal tissues	511	13949
fLung	Fetal Lung	Fetal tissues	526	17426
AoAF	Aortic Fibroblast	Stromal cells	521	14446
HPF	Pulmonary Fib.	Stromal cells	520	14323
IMR90	Fetal Lung Fib.	Stromal cells	511	10979
NHLF	Lung Fib.	Stromal cells	520	14323
NHDF_Ad	Adult Dermal Fib.	Stromal cells	521	13300
NHDF_Neo	Neonatal Dermal Fib.	Stromal cells	514	15177
HCM	Cardiac Fib.	Stromal cells	519	14804
HCF	Cardiac Fib.	Stromal cells	515	14158
HPAF	Pulmonary Artery Fib.	Stromal cells	524	13164
AG10803	Skin Fib.	Stromal cells	514	12166
HVMF	Mesenchymal Fib.	Stromal cells	520	14880
HMF	Mammary Fib.	Stromal cells	519	13611
HPdLF	Periodontal Fib.	Stromal cells	513	12570
HFF	Foreskin Fib.	Stromal cells	506	11796
HA-h	Hippocampal Astrocyte	Visceral cells	523	15956
HSMM	Skeletal Myoblast	Visceral cells	514	13394
SKMC	Skeletal Muscle	Visceral cells	522	16840
NH-A	Astrocyte	Visceral cells	510	9033
SK-N-SH_RA	Neuroblastoma	Cancer	501	12449
HepG2	Hepatoblastoma	Cancer	488	12546
H7-hESC	Embryonic Stem Cells	Embryonic Stem Cells	526	16068
